# COVID-19 Vaccine Effectiveness of Booster Doses Against Delta and Omicron Variants Over Follow-up Times Using Longitudinal Meta-analysis

**DOI:** 10.34172/jrhs.2024.161

**Published:** 2024-09-30

**Authors:** Farideh Mostafavi, Mansour Bahardoust, Francesco Sera, Alireza Amirabadizadeh, Sepehr Allahyari, Paddy Ssentongod, Manochehr Karami, Seyed Saeed Hashemi Nazari

**Affiliations:** ^1^Student Research Committee, School of Public Health and Safety, Shahid Beheshti University of Medical Sciences, Tehran, Iran; ^2^Department of Epidemiology, School of Public Health and Safety, Shahid Beheshti University of Medical Sciences, Tehran, Iran; ^3^Department of Statistics, Computer Science and Applications ‘G.Parenti’, University of Florence, Florence, Italy; ^4^Endocrine Research Center, Research Institute for Endocrine Sciences, Shahid Beheshti University of Medical Sciences, Tehran, 9717113163, Iran; ^5^Department of Virology, Doctor of Veterinary Medicine Student, Faculty of Veterinary Medicine, Garmsar Branch, Islamic Azad University, Tehran, Iran; ^6^Department of Medicine, Penn State Milton S. Hershey Medical Center, Hershey, PA 17033, USA

**Keywords:** COVID-19 vaccines, Vaccine efficacy, COVID-19 vaccine booster shot

## Abstract

**Background:** COVID-19 is a viral disease caused by the *SARS-CoV-2*, leading to several variants. This study aimed to examine the effectiveness of booster doses against the Delta and Omicron variants over different follow-up times.

**Study Design:** This was a longitudinal meta-analysis.

**Methods:** Searches were performed in PubMed, Cochrane Library, Scopus, and Web of Science databases, and eighty studies were selected for investigation. The analyses were separately performed on the unvaccinated control group (UNVCG) and the complete two doses of the vaccine control group (C2DCG) against Delta and Omicron variants. Three outcomes were examined, including symptomatic infection, hospitalization, and death.

**Results:** Vaccine effectiveness (VE) in UNVCG studies for symptomatic infection revealed a non-linear trend against Omicron with a peak of 67.3%, declining to 27.1% after 25 weeks after a booster dose. The mean of VE for hospitalization over time started to decrease after four weeks against Omicron and after eight weeks against Delta. The VE reached a peak at week eight (96.0%) and started to decline with a VE of 93.3% after 20 weeks after the booster dose against Delta. It was 90.8% at week four and decreased to 73.4% after 25 weeks after the booster dose against Omicron. VE in the C2DCG studies demonstrated more decreases in outcomes over time.

**Conclusion:** Our findings showed a tendency to decrease effectiveness over time based on outcomes and variants. The early protection levels were lower in Omicron. Moreover, the VE decrease over time was stronger in Omicron compared to the Delta variant.

## Background

 The main strategy of the health systems for controlling the acute stage of the COVID-19 epidemic was universal vaccination.^1^ Since the beginning of the epidemic, global efforts have focused on the development of safe and effective vaccines to prevent COVID-19. Before the epidemic, the development of a vaccine was a long and complex process that lasted several decades before the approval of its clinical use. Shortly after the onset of COVID-19, scientists began a race to develop an effective and safe vaccine against this disease based on new and old vaccine technologies. In less than two years, more than 300 vaccines worldwide were candidates, and 117 vaccines were developed in different clinical stages, of which 30 vaccines are in phase three studies. As of mid-2021, seven COVID-19 vaccines have been licensed for emergency use.^1,2^

 Effectiveness data show high levels of short-term protection of COVID-19 vaccines against clinical diseases and severe outcomes, including hospitalization and death.^3-5^ However, there is evidence that protection against the disease decreases with time and against different virus variants.^6^ In late 2020 and 2021, a wide range of SARS-CoV-2 variants emerged, replacing the original Wuhan strain, some of which were associated with increased transmission and successive waves of infection in many countries.

 Due to the decrease in the effectiveness of vaccines after the second dose and the occurrence of different variants of the virus, booster dose vaccination started in several countries. Studies are being implemented to evaluate the effectiveness of the third dose or booster in these countries.^7^

 This study aims to integrate the published data regarding the effectiveness of the third dose or first booster vaccines to prevent symptomatic, severe disease, and death with a multivariate meta-analysis method using the main outcome set for the effectiveness of COVID-19 vaccines in clinical trials and observational studies that have been conducted so far. Our results may provide additional evidence-based information to help select the best policy to achieve increased coverage of booster dose vaccination against COVID-19 and reduce severe disease and mortality.

## Methods

###  Search strategy

 PubMed, the Cochrane Library, EMBASE, Scopus, and the Web of Science databases were searched from January 1, 2020, to March 25, 2023, following the Preferred Reporting Items for Systematic Reviews and Meta-Analyses. Three authors independently researched the databases, reviewed the relevant titles and summaries, and extracted the information required for data analysis. The authors and other experts were contacted for inadequate data.

 The general search strategy included (((COVID 19[Title/Abstract] ) OR (SARS CoV 2 Infection[Title/Abstract])) OR (Coronavirus Disease 2019[Title/Abstract])) OR (COVID-19 Pandemic[Title/Abstract]) AND ((COVID-19 Vaccines[Title/Abstract]) OR (SARS-CoV-2 Vaccines[Title/Abstract])) OR (Coronavirus Disease-19 Vaccine[Title/Abstract]) AND ((Vaccine Efficacy[Title/Abstract]) OR (Vaccine effectiveness[Title/Abstract])) AND ((booster dose[Title/Abstract]) OR (booster shot[Title/Abstract])) OR (vaccine booster[Title/Abstract]).

 All the retrieved studies were stored in databases using EndNote. Duplicate studies were removed using the automatic removal function of EndNote. In addition, for more certainty, some remaining repeated studies were removed manually. Unpublished studies, letters, or short communication were not included in the investigation.

###  Study selection

 After screening the titles, the articles’ abstracts and full texts were reviewed until the studies that met the inclusion criteria were included in the study. Studies were included only if the booster dose and the two primary doses were specified in the articles, and the effectiveness of the booster dose vaccine was calculated. Clinical trials or observational studies that included the inclusion criteria and had sufficient data on the effectiveness of the third vaccine dose at defined time points against the Delta or Omicron variant were included in the study. On the other hand, studies that only examined the effectiveness of the first and second doses were excluded from the investigation.

###  Data extraction

 Two researchers independently extracted and tabulated the key data from the studies. A third investigator was consulted if there was disagreement. The articles were initially screened by two independent researchers, and any differences in the selection of articles in the screening process were resolved in consultation with the third researcher. The extracted variables were the name of the first author, type of study, year of publication, sample size, the type of previous second dose and first booster dose, country, age, time since the injection of the booster vaccine dose (in the week), previous COVID-19 infection, dominant variant, and vaccine efficacy or vaccine effectiveness (VE).

###  Quality assessment

 The quality or risk assessment of the cohort and case-control studies was performed by the Newcastle-Ottawa scale.^8^ This checklist evaluates the quality of studies in the selection, comparability, and outcome/exposure sections and assigns a star(s) for each item.^8^

 The methodological quality and risk of bias of randomized controlled trial (RCT) studies were evaluated using the revised Cochrane risk-of-bias tool for randomized trials (RoB 2).^9^ This involved assessing random sequence generation, allocation concealment, blinding of participants and healthcare personnel, blinded outcome assessment, completeness of outcome data, selective reporting, and other biases. Based on this tool, the studies were categorized into three levels of quality (low, some concerns, and high).

###  Statistical analysis

 The effectiveness of the booster dose (third dose) was investigated for seven types of vaccines, including BNT162b2, Moderna, ChAdOx1, CoronaVac, Jansen, mRNA, and Sinopharm. The comparison group to calculate the VE was different in studies; in some studies, booster vaccination was compared with the complete two doses of the vaccine control group (C2DCG), and in other studies, booster vaccination was compared with the unvaccinated control group (UNVCG). Accordingly, analyses were performed separately in terms of the two control groups for Delta and Omicron variants. Three outcomes were investigated, including VE for symptomatic COVID-19 infection, COVID-19-related hospitalization, and COVID-19-related death.

 In this review, some studies reported that the VE for deaths was 100% while not reporting the confidence interval (CI) and standard error. Firth’s regression was used to calculate the variance of these studies to include them in the analysis since there were zero deaths in the vaccine group, and the sample size was extremely small in these studies.

 The results of each study were pooled at each time point to provide overall VE. Subgroup analyses were performed to identify heterogeneity sources across studies. In addition, the univariate meta-analysis random-effects model was implemented, allowing studies to have their own population effect sizes, according to the type of booster (BNT162b2, Moderna, ChAdOx1, CoronaVac, Jansen, mRNA, and Sinopharm), age (≥ 12 years old and ≥ 50 years old), study design (RCT, case control, negative case control, and cohort), previous COVID-19 infection (yes/no), and risk of bias (low, moderate, or high risk).

 The univariate model assumes that the effect sizes are independent at different time points, so they ignore the correlation between effect sizes, and this can increase the standard error of the point estimates. To evaluate the VE over the follow-up period (1, 4, 8, 12, 16, 20, 24, and > 25 weeks), a longitudinal meta-analysis was performed, which assumes that the effect sizes at one time are correlated with the effect sizes at other times. The longitudinal structure of the meta-analysis at different time points, using a multivariate approach, can be defined as follows:

*y*_i_*= X*_i_*β*_1_*+ b*_i_* + ε*_i_

 which is a general linear mixed model. β1 is the vector of fixed effects at each follow-up time, and *b*_i _denotes the vector of random effects at each follow-up time. Further, *ε*_i_ represents the vector of residuals. In the multivariate model, random effects have a multivariate normal distribution with a zero mean and a covariance matrix D.^10,11^ In this analysis, the structure of the covariance matrix was considered autoregressive (AR), which is an extension of the independent random time effects model, where the dependence between effect sizes is measured by the dependence between random time effects. This model considers a heterogeneous AR(1) covariance structure for random time effects while assuming within-study serial correlations between longitudinal effect sizes, Si = diag ( ${ó}_{i1}^{2},...,\;{ó}_{i8}^{2}$ ). Consequently, the variance-covariance matrix is V(Yi) = ∑ + Si, diagonal elements ( $\tau_{1}^{2}+{ó}_{i1}^{2},...,\;\tau_{8}^{2}+{ó}_{i8}^{2}$ ), and off-diagonal elements ( $\rho_{\tau }^{\left|t-t'\right|}\tau_{t}\tau_{t'\;}$ ). For time points t and t' , ρτ is the correlation between any two adjacent random time effects.^10^ Therefore, the dependence between effect sizes increases with a decrease in the distance between them. This is appropriate in longitudinal studies where the loss of follow-up increases over time, so that the effect values measured more distant are less dependent than those measured closer together.

 Publication bias was evaluated graphically with funnel plots and by the trim-and-fill, Egger’s test, and Bagg’s test. Trim-and-fill is a non-parametric approach that assesses the asymmetry of the funnel plot. The horizontal axis of the funnel plot indicates the effect sizes of studies, and the vertical axis denotes their measures of precision (e.g., standard errors, sample size, and the like). First, this method figures out the publication bias by detecting any asymmetry in funnel plots, then adjusts the bias by trimming or removing small studies, causing asymmetry in the plot to estimate the true center of the plot, and finally fills or replaces the removed studies and their missing ‘counterparts’ around the center.^12^

 Egger’s test is based on a linear regression of the studies’ effect size on their standard errors or any other precision measures to quantify funnel plot asymmetry, in which a significant intercept indicates possible publication bias. Bagg’s test uses Kendall’s tau to evaluate whether there is a significant correlation between the ranks of studies’ effect size and the rank of their variance. The significance of Bagg’s test shows no selection bias present, and non-significant studies are less likely to be published ^13^. Meta-analysis was performed with Stata 17 and R software with the metafor package in R (version R-4.2.3).^11^

## Results

**Figure 1:**
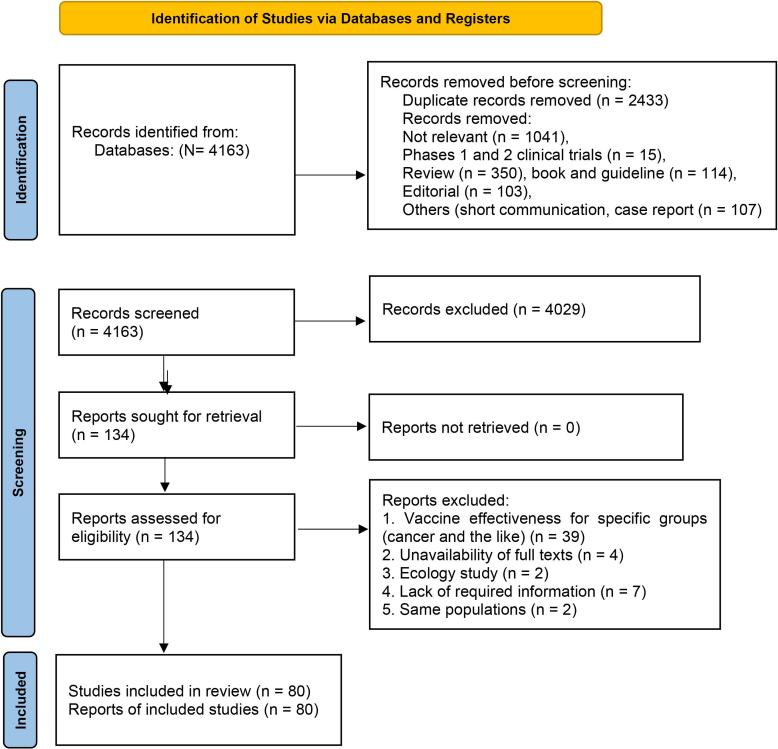
PRISMA flow diagram for systematic reviews of vaccine effectiveness/effectiveness against SARS-CoV-2 infection. *Note*. PRISMA: Preferred Reporting Items for Systematic reviews and Meta-Analyses; SARS-CoV-2: Severe acute respiratory syndrome coronavirus 2

 Overall, 4163 studies from databases were identified for analysis. After removing duplicates, 1,730 studies were screened for eligibility in terms of abstracts and titles. After removing 1596 studies, 134 studies were checked in terms of full texts, and finally, 80 studies were included in the analysis (Figure 1). Out of the 80 studies, there were two RCT studies,^14,15^ eight case-control studies,^16-23^ 33 negative case-control studies,^24-56^ and 37 cohort studies^57-93^ from 28 countries (Figure 1).

 The mean ± standard deviation (SD) follow-up times since the booster dose in studies with the UNVCG were 12.6 (± 0.6) and 8.0 (± = 0.3) weeks for the Omicron and Delta variants, respectively. In studies with the complete vaccinated control group, the mean follow-up times after the booster dose were 13.7 (± 0.7) and 7.2 (± 0.5) weeks for the Omicron and Delta variants, respectively.

 Among the 80 studies, 18 reported VE at only one time point. These studies were separately analyzed and excluded from the VE time trend analysis over the follow-up period. In addition, 11 of these 18 studies were UNVCG, with a mean follow-up time of 20.1 weeks (SD = 1.4) against the Omicron variant and 15.6 weeks (SD = 1.9) against the Delta variant. In 11 studies with UNVCG, the pooled VE against the Omicron variant was 73.2% (95% CI: 52.6–84.8) for the symptomatic infection and 86.5% (95% CI: 77.0–92.1) for hospitalization. The pooled VE against the Delta variant was 75.7 (95% CI: 73.3–77.8) and 92.5 (95% CI: 89.0–96.9) for symptomatic infection and hospitalization, respectively.

 The other 7 out of 18 studies were C2DCG, with a mean follow-up time of 35.1 weeks (SD = 2.9) and 17.7 weeks (SD = 1.9) against the Omicron and Delta variants, respectively. In the seven studies with C2DCG, the pooled VE against the Omicron variant was 68.5 (95% CI: 58.9–75.8) for the symptomatic infection and 85.2 (95% CI: 72.7–92.0) for hospitalization. Moreover, the pooled VE against the Delta variant was 91.9 (95% CI: 88.5–94.3) and 86.7 (95% CI: 64.5–95.0) for symptomatic infection and hospitalization, respectively. There were not enough studies with death as the outcome to evaluate VE.

###  Overall pooled vaccine effectiveness 

*VE in studies with the UNVCG*: The overall VE (across all time points) for symptomatic infection was 54.0% (95% CI: 51.0–57.0) and 91.0% (95% CI: 90.0–92.0) against the Omicron and Delta variants, respectively (Supplementary file 1, Figures S1 and S2). The VE for hospitalization was 74.0% (95% CI: 65.0–80.0) and 92.0% (95% CI: 90.0–94.0) (Supplementary file 1, Figures S3 and S4). Additionally, the VE for death was 80.0 (95% CI: 57.0–91.0) and 85.0 (95% CI: 78.0–90.0) against the Omicron and Delta variants, respectively (Supplementary file 1, Figures S5 and S6).

*VE in studies with the UNVCG*:The VE for symptomatic infection was 44.0% (95% CI: 40.0–48.0) and 86.0% (95% CI: 82.0–89.0), and it was 68.0% (95% CI: 63.0–72.0) and 90.0% (95% CI: 82.0–94.0) for hospitalization against the Omicron and Delta variants, respectively. There were not enough studies with death as the outcome for VE evaluation.

###  Vaccine effectiveness trend in studies with the unvaccinated control group

*Symptomatic infection*: Multivariate and univariate meta-analyses showed a tendency toward a decrease in long-term VE for symptomatic infection. The mean VE against Delta reached a peak of 91.8% (95% CI: 87.5–94.6) after eight weeks and started to decline with a VE equal to 76.5% (95% CI: 39.0–90.9) after 24 weeks after the booster dose. Furthermore, the effectiveness of the booster dose against the Omicron variant demonstrated a non-linear trend with a peak of 67.3% (95% CI: 58.1–74.4) after eight weeks and decreased to 27.1% (95 CI: 23.9–30.1) after 25 weeks after the booster dose (Table 1).

**Table 1:** Meta-analysis results for COVID-19 vaccine effectiveness^*^

**Time since booster dose**	**Time 1** **(1 week)**	**Time 4** **(4 weeks)**	**Time 8** **(8 weeks)**	**Time 12** **(12 weeks)**	**Time 16** **(16 weeks)**	**Time 20** **(20 weeks)**	**Time 24** **(24 weeks)**	**Time 25** **(25≥weeks)**
Symptomatic infection in the Delta predominant period (95% CI)								
Univariate analysis	78.0 (67.0, 85.0)	92.0 (90.0, 94.0)	95.0 (94.0, 96.0)	93.0 (92.0, 94.0)	90.0 (89.0, 91.0)	85.0 (73.0, 91.0)	79.0 (47.0, 92.0)	
Multivariate analysis	66.0 (45.4, 78.8)	90.0 (86.6, 92.5)	91.8 (87.5, 94.6)	90.1 (86.1,92.9)	89.6 (89.1, 90.1)	80.1 (77.3, 82.5)	76.5 (39.0, 90.9)	
Number of studies	18	26	20	28	18	5	2	
Hospitalization								
Univariate analysis	92.0 (88.0, 94.0)	93.0 (88, 96)	94.0 (88.0, 97.0)	93 (87.0, 96.0)	89.0 (76.0, 95.0)	87.0 (78.0, 92.0)		
Multivariate analysis	82.0 (72.4, 88.2)	94.8 (92.2, 96.5)	96.0 (93.3, 97.6)	96.0 (93.5, 97.5)	96.9 (94.1, 98.4)	93.3 (89.2, 95.9)		
Number of studies	11	14	16	17	11	5		
Death								
Univariate analysis	90.0 (83.0, 94.0)	90.0 (80.0, 95.0)	81.0 (49.0, 93.0)	83.0 (51.0, 94.0)	62.0 (-40, 86.0)	71.0 (30.0, 88.0)		
Multivariate analysis	79.9 (61.5, 89.5)	94.1 (89.4, 96.8)	95.1 (89.0, 97.8)	96.2 (88.8, 98.7)	88.0 (67.8, 95.6)	90.6 (74.1, 96.6)		
Number of studies	6	10	12	13	7	4		
Symptomatic infection in the Omicron predominant period (95% CI)								
Univariate analysis	53.0 (41.0, 63.0)	61.0 (57.0, 65.0)	58.0 (49.0, 65.0)	56.0 (51.0, 62.0)	46.0 (33.0, 57.0)	56.0 (52.0, 59.0)	46.0 (43.0, 49.0)	29.0 (22.0, 35.0)
Multivariate analysis	45.4 (22.7, 61.4)	62.1 (56.7, 66.8)	67.3 (58.1, 74.4)	59.0 (49.8, 66.4)	55.4 (41.9, 65.8)	42.7 (27.4, 54.8)	46.1 (43.5, 48.5)	27.1 (23.9, 30.1)
Number of studies	12	19	18	28	10	4	2	11
Hospitalization								
Univariate analysis	85.0 (3.0, 98.0)	89.0 (83.0, 93.0)	86.0 (77.0, 91.0)	81.0 (54.0, 92.0)	53.0 (-54.0, 86.0)	77.0 (-4.0, 95.0)	52.0 (-13.0, 80.0)	45.0 (22.0, 61.0)
Multivariate analysis	60.9 (5.7, 83.8)	90.8 (85.7, 94.0)	87.9 (79.0, 93.0)	81.1 (67.0, 89.2)	88.2 (75.4, 94.4)	81.9 (66.6, 90.2)	80.7 (63.1, 89.9)	73.4 (51.6, 85.4)
Number of studies	2	6	12	8	3	5	4	16

*Note*. CI: Confidence interval; COVID: Coronavirus disease. ^*^The VE of COVID booster dose vaccine compared to the unvaccinated group by endpoints (Outcomes).

*Hospitalization:* The mean of VE over time in the delta period looks more stable than in the omicron variant period. The mean of VE reached a peak of 96.0% (95% CI: 93.3–97.6) after eight weeks and started to decrease with a VE equal to 93.3% (95% CI: 89.2–95.9) after 20 weeks after the booster dose against Delta. In the Omicron period, the univariate and multivariate models had different estimates. Multivariate models showed a VE of 90.8% (95% CI: 85.7–94.0) against the Omicron variant at four weeks and decreased to 73.4% (95% CI: 51.6–85.4) after 25 weeks after the booster dose (Table 1).

*Death*: For death, multivariate results revealed a more stable trend, which was 95.1% (95% CI: 89.0–97.8) after eight weeks and then reached a VE equal to 90.6% (95% CI: 74.1–96.6) after 20 weeks after the booster dose (Table 1). There were insufficient studies with death as a result to estimate VE time trends relative to Omicron.

###  Vaccine effectiveness trend in studies with complete two doses of the vaccine control group

*Symptomatic infection*: The results indicated a tendency for a decrease in the long-term effectiveness of the vaccine for the studied outcomes (Table 2). For example, a multivariate meta-analysis showed a decreasing trend of 58.7% (95% CI: 53.5–63.3) for the VE against the Omicron variant after four weeks, which declined to 19.1% (95% CI: 6.0–30.3) after 25 weeks after the booster dose.

**Table 2:** Meta-analysis results for vaccine effectiveness^*^

**Time Since the Booster Dose**	**Time 1 (1 week)**	**Time 4 (4 weeks)**	**Time 8 (8 weeks)**	**Time 12 (12 weeks)**	**Time 16 (16 weeks)**	**Time 20 (20 weeks)**	**Time 24 (24 weeks)**	**Time 25 (≥25 weeks)**
Symptomatic infection in the Delta predominant period (95% CI)								
Univariate analysis	80.0 (60.0, 90.0)	84.0 (67.0, 92.0)	91.0 (85.0, 95.0)	85.0 (80.0, 88.0)	88.0 (81.0, 92.0)			
Multivariate analysis	36.1 (-26.6, 67.7)	77.1 (59.5, 87.0)	91.4 (85.3, 95.0)	81.9 (74.5, 87.1)	70.5 (51.1, 82.2)			
Number of studies	12	15	4	22	5			
Hospitalization								
Univariate analysis	87.0 (81.0, 91.0)	90.0 (78.0, 96.0)	89.0 (80.0, 94.0)	89.0 (-28.0, 99.0)				
Multivariate analysis	81.9 (69.2, 89.3)	81.6 (52.6, 92.8)	89.3 (80.2, 94.3)	84.1 (8.8, 97.2)				
Number of studies	1	4	2	2				
Symptomatic infection in the Omicron predominant period (95% CI)								
Univariate analysis	63.0 (55.0-70.0)	58.0 (53.0, 63.0)	45.0 (41.0, 50.0)	50.0 (43.0, 56.0)	37 (30.0, 44.0)	21.0 (16.0, 25.0)	31.0 (21.0, 40.0)	5.0 (-7.0, 16.0)
Multivariate analysis	55.2 (44.7, 63.8)	58.7 (53.5, 63.3)	51.2 (46.4, 55.6)	47.1 (39.5, 53.8)	37.2 (29.9, 43.7)	26.2 (21.7, 30.5)	31.5 (21.9, 40.0)	19.1 (6.0, 30.3)
Number of studies	8	20	30	36	9	7	8	19
Hospitalization								
Univariate analysis	39.0 (2.0, 62.0)	76.0 (65.0, 83.0)	76.0 (69.0, 82.0)	61.0 (54.0, 68.0)	53.0 (36.0, 66.0)	82.0 (69.0, 90.0)	67.0 (52.0, 77.0)	62.0 (47.0, 73.0)
Multivariate analysis	31.6 (-12.2, 58.3)	73.0 (61.6, 81.1)	72.6 (65.2,78.4)	66.7 (71.5, 61.0)	60.7 (54.2, 66.3)	67.9 (58.2, 75.3)	66.6 (45.3, 79.7)	49.1 (25.4, 65.3)
Number of studies	3	5	12	11	5	6	3	12

*Note*. COVID: Coronavirus disease; CI: Confidence interval; ^*^ The COVID booster dose vaccine compared to the complete vaccinated group.

*Hospitalization*: Based on the results, there was a decrease in VE over time for hospitalization (Table 2); for example, it reached a peak of 73.0% (95% CI: 61.6–81.1) against Omicron at week eight and decreased to 49.1% (95% CI: 35.4–65.3) after 25 weeks after the booster dose in multivariate analyses. A few studies reported a VE of 84.1% (95% CI: 8.8–97.2) against the Delta variant for the first 12 weeks.

*Death*: The outcome of death in the complete vaccinated control group was not analyzed due to the small number of studies.

###  Publication bias

 Publication bias was not found with Begg’s test, but Egger’s tests demonstrated publication bias in the analysis of VE in some subgroups; no significant changes were observed after using the trim-and-fill method.

## Discussion

 This review focused on investigating the effectiveness of the booster dose (third dose) of COVID-19 vaccines in terms of the two control groups for the outcomes of death, hospitalization, and symptomatic infection separately in periods that each variant of Omicron or Delta was predominant.

 Based on the results of our study, the overall VE of the booster dose against the Omicron variant for the outcomes of symptomatic infection and hospitalization was 54% and 74% in the UNVCG, as well as 44% and 68% in the completed vaccinated control group, respectively. It was found that the VE of the booster dose for the outcomes of symptomatic infection and hospitalization was higher in studies with the UNVCG compared to the complete vaccinated control group. It was expected that the additive effect of the booster dose on previous vaccination would to be more obvious when the comparison group was unvaccinated, and the obtained data confirmed this issue.

 The overall VE of the booster dose against Delta for the outcomes of death, hospitalization, and symptomatic infection in the non-vaccinated control group was 85.0%, 92.0%, and 91.0%, respectively, and in the complete vaccinated control group, it was 86.0% and 90.0% for hospitalization and symptomatic infection. In studies with the complete vaccinated control group, previous vaccination provided some prevention against the outcomes, and the effectiveness of the vaccine in the booster arm compared to this control group was lower in comparison with the studies with the UNVCG.

 This review revealed that the VE reached its peak 4 and 8 weeks after the booster dose for the studied outcome against Omicron and Delta, respectively, and then the VE started to decrease. Our findings demonstrated a small but significant decrease in VE over the follow-up periods against the Delta variant, but this downward trend was significantly stronger against the Omicron variant, especially for symptomatic infection and hospitalization in both control groups. These results are in line with those of the study by Patalon et al,^45^ showing that in the Omicron variant, the VE against infection decreased from 53.4% four weeks after vaccination to 16.5% twelve weeks after vaccination. This downward trend can be justified considering the nature of this type of variant, whose its mutagenic and contagious abilities can change over time. Rana et al^94^ reported that the Omicron variant has a significant amount of mutation and can have a higher transmission rate than the other variants, which can impair the effectiveness of diagnostic equipment for previous variants and the effectiveness of vaccination. Yu et al^95^ estimated the time-varying transmissibility and the relative transmissibility of Beta, Delta, and Omicron variants. They found that the transmissibility of the Omicron variant was clearly greater than that of the other two variants.

 According to the criteria recommended by the World Health Organization, the adequate effectiveness response of the COVID-19 vaccine indicated at least 70.0% against symptomatic infection and at least 90.0% against hospitalization and death.^96,97^ Our results are consistent with those of the study performed by Wu et al,^97^ indicating the lack of effectiveness of the vaccine against the Omicron variants in the UNVCG for symptomatic infection, but the reported effectiveness in our review reached the recommended value of 90.0% at the peak of VE after 4 weeks and then fell below the recommended value in all follow-up periods in the long term. In the completed control group, the recommended criteria were not met for the studied outcomes against Omicron. The recommended effectiveness for all outcomes in the two control groups was observed in the Delta variant.

 The present study has some limitations that should be mentioned. Firstly, although the researchers attempted to control the amount of heterogeneity in this study with different methods, there was still a significant amount of heterogeneity in our study, which is probably due to the existence of variables that were influential in heterogeneity between studies and were not considered in our study. Secondly, the included studies in this meta-analysis review were different in terms of the study setting, different periods, locations, and populations, and the real overall effect could have been affected by this heterogeneity. Thirdly, some variables, such as previous COVID-19 infection and age, were not well-defined in the studies or were missing, and the authors did not cooperate in sharing information; thus, we could not perform adjusted multivariate analysis to estimate VE by controlling variables. Fourth, many outcomes, especially symptomatic infection, have been reported in some studies based on self-reported forms, which could have affected the overall effect on the results. Finally, we could not assess the role of the different sub-variants of Omicron due to a lack of sufficient studies or reports on the sub-variants of Omicron in different studies.

**Highlights**

- The mean of VE for hospitalization over time started to decrease after four and eight weeks against Omicron and Delta, respectively.
- The VE reached a peak after eight weeks and began to decline with a VE after 20 weeks after the booster dose against Delta.
- The VE reached a peak after four weeks and started to decline with a VE after 25 weeks after the booster dose against Omicron.
- The early protection levels were lower in Omicron variants, and the VE decrease over time was stronger in the Omicron variant in comparison to the Delta variant.

## Conclusion

 Our study confirmed a tendency to decrease effectiveness over time based on outcomes and variants. The early protection levels were lower in Omicron variants, and the VE decrease over time was stronger in the Omicron variant in comparison to Delta.

## Authors’ Contribution

**Conceptualization:** Farideh Mostafavi, Mansour Bahahrdoust, and Seyed Saeed Hashemi Nazari.

**Data curation:** Farideh Mostafavi, Mansour Bahahrdoust, and Alireza Amirabadizadeh.

**Formal analysis:** Farideh Mostafavi, Fransisco Sera, Manochehr Karami, and Seyed Saeed Hashemi Nazari.

**Investigation:** Manochehr Karami and Seyed Saeed Hashemi Nazari.

**Methodology:** Farideh Mostafavi, Francesco Sera, and Seyed Saeed Hashemi Nazari.

**Project administration:** Seyed Saeed Hashemi Nazari.

**Software:** Farideh Mostafavi, Francesco Sera, Paddy Ssentongod, and Seyed Saeed Hashemi Nazari.

**Supervision:** Seyed Saeed Hashemi Nazari.

**Validation:** Manochehr Karami and Seyed Saeed Hashemi Nazari.

**Visualization:** Manochehr Karami and Seyed Saeed Hashemi Nazari.

**Writing–original draft:** Farideh Mostafavi, Mansour Bahahrdoust, and Alireza Amirabadizadeh.

**Writing–review & editing:** Manochehr Karami, Sepehr Allahyari, and Seyed Saeed Hashemi Nazari.

## Competing Interests

 The authors declare no competing interests.

## Ethical Approval

 Not applicable.

## Funding

 Not applicable.

## Supplementary Files

Supplementary file 1 contains Figures S1-S6.
