# Supplementary material for: COVID-19 Vaccine Effectiveness of Booster Doses Against Delta and Omicron Variants Over Follow-up Times Using Longitudinal Meta-analysis
Source: J Res Health Sci. 2024 Sep 30;24(4):e00626. doi: 10.34172/jrhs.2024.161 (PMC11492529; doi:10.34172/jrhs.2024.161)
Supplement: Supplementary file 1 — contains Figures S1-S6. [file jrhs-24-e00626-s001.pdf]

JRHS
Journal of Research in Health Sciences
doi:10.34172/jrhs.2024.161
JRHS 2024; 24(4):e00626

Figure S1 :The vaccine effectiveness against Omicron variant for the symptomatic infection by variables in the unvaccinated control group

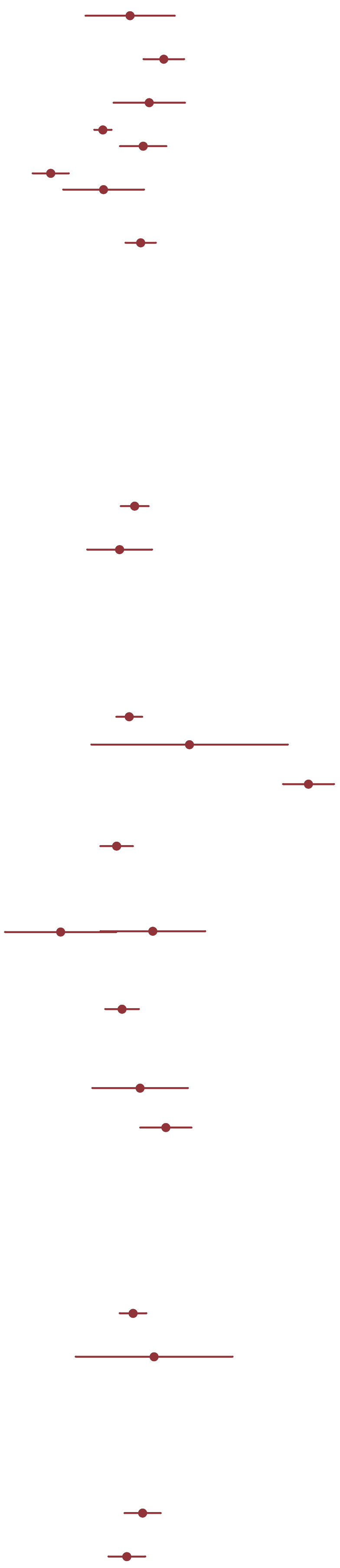

Study	K	Effectiveness with 95% CI	p-value
**time**			
1	12	0.53 [0.41, 0.63]	0.000
4	19	0.61 [0.57, 0.65]	0.000
8	18	0.58 [0.49, 0.65]	0.000
12	28	0.56 [0.51, 0.62]	0.000
16	10	0.46 [0.33, 0.57]	0.000
20	4	0.56 [0.52, 0.59]	0.000
24	2	0.46 [0.43, 0.49]	0.000
25	11	0.29 [0.22, 0.35]	0.000
Test of group differences: $Q_b(7) = 98.47$, $p = 0.00$			
**age**			
>=12 yr	96	0.54 [0.51, 0.58]	0.000
>50yr	8	0.50 [0.41, 0.58]	0.000
Test of group differences: $Q_b(1) = 0.70$, $p = 0.40$			
**rob**			
low	98	0.53 [0.50, 0.56]	0.000
moderate	5	0.66 [0.43, 0.80]	0.000
high	1	0.82 [0.79, 0.84]	0.000
Test of group differences: $Q_b(2) = 151.28$, $p = 0.00$			
**booster**			
Any	16	0.59 [0.45, 0.69]	0.000
BNT162b2	46	0.50 [0.45, 0.54]	0.000
ChAdOx1	11	0.51 [0.47, 0.55]	0.000
CoronaVac	1	0.32 [0.09, 0.50]	0.010
mRNA	11	0.56 [0.43, 0.66]	0.000
moderna	19	0.61 [0.56, 0.66]	0.000
Test of group differences: $Q_b(5) = 17.31$, $p = 0.00$			
**studydesign**			
negative case control	97	0.54 [0.50, 0.57]	0.000
cohort	7	0.59 [0.37, 0.73]	0.000
Test of group differences: $Q_b(1) = 0.27$, $p = 0.60$			
**previouscovidinfection**			
no	44	0.56 [0.52, 0.60]	0.000
yes	60	0.52 [0.47, 0.57]	0.000
Test of group differences: $Q_b(1) = 1.44$, $p = 0.23$			

Overall

Heterogeneity: $\tau^2 = 0.12$, $I^2 = 99.70\%$, $H^2 = 334.38$

Test of $\theta_i = \theta_j$: $Q(103) = 30484.85$, $p = 0.00$

Random-effects REML model

0.54 [0.51, 0.57] 0.000

0.86

0.00 0.39 0.63 0.78

Figure S2: The vaccine effectiveness against Delta variant for the symptomatic infection by variables in the unvaccinated control group

Study	K	Effectiveness with 95% CI	p-value
time			
1	18	0.78 [0.67, 0.85]	0.000
4	26	0.92 [0.90, 0.94]	0.000
8	20	0.95 [0.94, 0.96]	0.000
12	28	0.93 [0.92, 0.94]	0.000
16	18	0.90 [0.89, 0.91]	0.000
20	5	0.85 [0.73, 0.91]	0.000
24	2	0.79 [0.47, 0.92]	0.001
Test of group differences: $Q_b(6) = 72.47$, $p = 0.00$			
**age**			
>=12 yr	80	0.92 [0.90, 0.93]	0.000
>50yr	37	0.91 [0.88, 0.93]	0.000
Test of group differences: $Q_b(1) = 0.54$, $p = 0.46$			
**rob**			
low	115	0.91 [0.90, 0.92]	0.000
moderate	1	0.90 [0.88, 0.92]	0.000
high	1	0.68 [0.16, 0.88]	0.020
Test of group differences: $Q_b(2) = 7.86$, $p = 0.02$			
**booster**			
Any	17	0.81 [0.70, 0.88]	0.000
BNT162b2	70	0.92 [0.91, 0.93]	0.000
ChAdOx1	8	0.88 [0.84, 0.90]	0.000
CoronaVac	1	0.78 [0.76, 0.80]	0.000
mRNA	3	0.88 [0.76, 0.95]	0.000
moderna	18	0.95 [0.93, 0.96]	0.000
Test of group differences: $Q_b(5) = 228.73$, $p = 0.00$			
**studydesign**			
case control	4	0.80 [0.53, 0.92]	0.000
negative case control	72	0.91 [0.89, 0.92]	0.000
cohort	41	0.93 [0.91, 0.94]	0.000
Test of group differences: $Q_b(2) = 5.60$, $p = 0.06$			
**previouscovidinfection**			
no	22	0.93 [0.91, 0.94]	0.000
yes	95	0.91 [0.89, 0.92]	0.000
Test of group differences: $Q_b(1) = 3.60$, $p = 0.06$			
**Overall**		0.91 [0.90, 0.92]	0.000

Heterogeneity: $\tau^2 = 0.55$, $I^2 = 99.53\%$, $H^2 = 212.44$

Test of $\theta_i = \theta_j$: $Q(116) = 30793.31$, $p = 0.00$

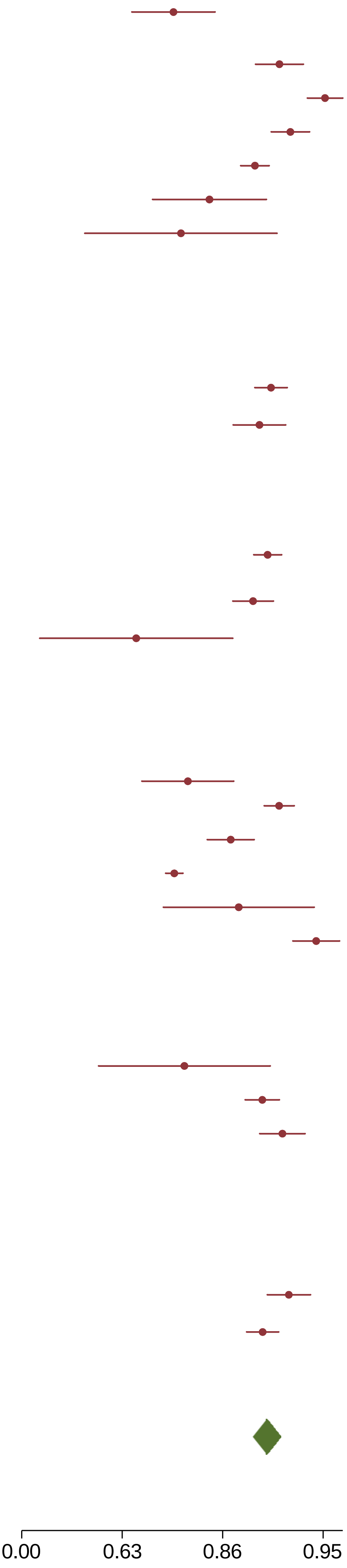

Random-effects REML model

Figure S3 : The vaccine effectiveness against Omicron variant for the hospitalization by variables in the unvaccinated control group

Study	K	Effectiveness with 95% CI	p-value
**time**			
1	2	0.85 [0.03, 0.98]	0.046
4	6	0.89 [0.83, 0.93]	0.000
8	12	0.86 [0.77, 0.91]	0.000
12	8	0.81 [0.54, 0.92]	0.000
16	3	0.53 [-0.54, 0.86]	0.211
20	5	0.77 [-0.04, 0.95]	0.056
24	4	0.52 [-0.13, 0.80]	0.093
25	15	0.45 [0.22, 0.61]	0.001
Test of group differences: Qb(7) = 43.31, p = 0.00			
**age**			
>=12 yr	36	0.75 [0.66, 0.82]	0.000
>50yr	19	0.70 [0.48, 0.83]	0.000
Test of group differences: Qb(1) = 0.33, p = 0.56			
**rob**			
low	51	0.73 [0.63, 0.80]	0.000
moderate	1	0.89 [0.87, 0.90]	0.000
high	3	0.78 [0.54, 0.89]	0.000
Test of group differences: Qb(2) = 29.76, p = 0.00			
**booster**			
Any	29	0.55 [0.33, 0.70]	0.000
BNT162b2	15	0.90 [0.87, 0.91]	0.000
mRNA	9	0.78 [0.68, 0.85]	0.000
moderna	2	0.79 [0.15, 0.95]	0.028
Test of group differences: Qb(3) = 48.05, p = 0.00			
**studydesign**			
case control	1	0.84 [0.83, 0.85]	0.000
negative case control	33	0.86 [0.81, 0.89]	0.000
cohort	21	0.25 [0.10, 0.37]	0.002
Test of group differences: Qb(2) = 265.50, p = 0.00			
**previouscovidinfection**			
no	14	0.89 [0.87, 0.91]	0.000
yes	41	0.64 [0.50, 0.74]	0.000
Test of group differences: Qb(1) = 42.80, p = 0.00			
**Overall**		0.74 [0.65, 0.80]	0.000
Heterogeneity: τ^2 = 1.10, I^2 = 100.00%, H^2 = 27345.44			
Test of $\theta_i = \theta_j$: Q(54) = 78771.53, p = 0.00			

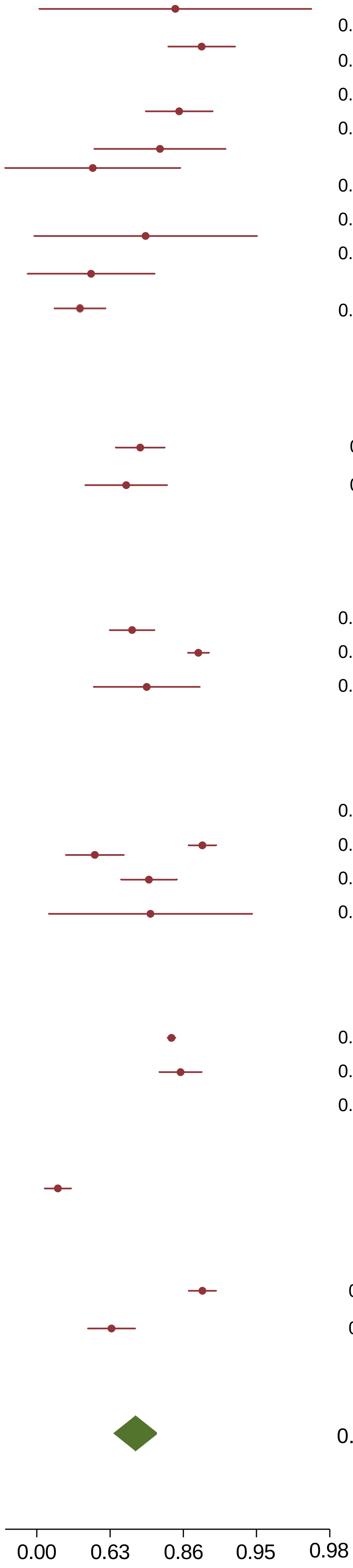

Random-effects REML model

Figure S4:The vaccine effectiveness against Delta variant for the hospitalization by variables in the unvaccinated control group

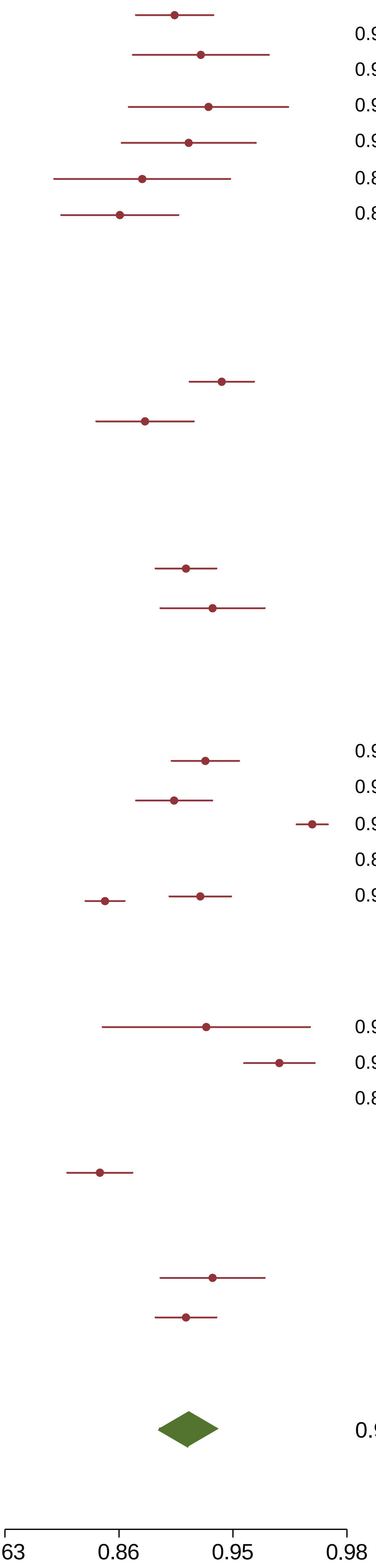

Study	K	Effectiveness with 95% CI	p-value
**time**			
1	11	0.92 [0.88, 0.94]	0.000
4	14	0.93 [0.88, 0.96]	0.000
8	16	0.94 [0.88, 0.97]	0.000
12	17	0.93 [0.87, 0.96]	0.000
16	11	0.89 [0.76, 0.95]	0.000
20	5	0.87 [0.78, 0.92]	0.000
Test of group differences: Qb(5) = 5.21, p = 0.39			
**age**			
>=12 yr	43	0.94 [0.93, 0.96]	0.000
>50yr	31	0.89 [0.83, 0.93]	0.000
Test of group differences: Qb(1) = 6.60, p = 0.01			
**rob**			
low	73	0.92 [0.90, 0.94]	0.000
moderate	1	0.94 [0.91, 0.96]	0.000
Test of group differences: Qb(1) = 0.74, p = 0.39			
**booster**			
Any	12	0.94 [0.91, 0.95]	0.000
BNT162b2	56	0.92 [0.88, 0.94]	0.000
ChAdOx1	3	0.98 [0.97, 0.98]	0.000
CoronaVac	1	0.85 [0.82, 0.87]	0.000
mRNA	2	0.93 [0.91, 0.95]	0.000
Test of group differences: Qb(4) = 273.32, p = 0.00			
**studydesign**			
case control	4	0.94 [0.84, 0.97]	0.000
negative case control	31	0.97 [0.95, 0.98]	0.000
cohort	39	0.84 [0.79, 0.88]	0.000
Test of group differences: Qb(2) = 53.56, p = 0.00			
**previouscovidinfection**			
no	1	0.94 [0.91, 0.96]	0.000
yes	73	0.92 [0.90, 0.94]	0.000
Test of group differences: Qb(1) = 0.74, p = 0.39			
**Overall**		0.92 [0.90, 0.94]	0.000

Heterogeneity: $\tau^2 = 1.03$, $I^2 = 95.49\%$, $H^2 = 22.16$

Test of $\theta_i = \theta_j$: $Q(73) = 1449.83$, $p = 0.00$

Random-effects REML model

Figure S5 : The vaccine effectiveness against Omicron variant for the death by variables in the unvaccinated control group

Study	K	Effectiveness with 95% CI	p-value
**time**			
12	3	0.80 [0.37, 0.94]	0.006
16	1	0.82 [0.80, 0.84]	0.000
Test of group differences: $Q_b(1) = 0.03$, $p = 0.86$			
**age**			
>=12 yr	2	0.89 [0.69, 0.96]	0.000
>50yr	2	0.64 [0.59, 0.69]	0.000
Test of group differences: $Q_b(1) = 4.79$, $p = 0.03$			
**rob**			
moderate	2	0.89 [0.69, 0.96]	0.000
high	2	0.64 [0.59, 0.69]	0.000
Test of group differences: $Q_b(1) = 4.79$, $p = 0.03$			
**booster**			
Any	3	0.72 [0.56, 0.82]	0.000
mRNA	1	0.94 [0.91, 0.96]	0.000
Test of group differences: $Q_b(1) = 21.92$, $p = 0.00$			
**studydesign**			
case control	1	0.94 [0.91, 0.96]	0.000
cohort	3	0.72 [0.56, 0.82]	0.000
Test of group differences: $Q_b(1) = 21.92$, $p = 0.00$			
**previouscovidinfection**			
no	1	0.94 [0.91, 0.96]	0.000
yes	3	0.72 [0.56, 0.82]	0.000
Test of group differences: $Q_b(1) = 21.92$, $p = 0.00$			
**Overall**		0.80 [0.57, 0.91]	0.000
Heterogeneity: $\tau^2 = 0.65$, $I^2 = 98.42\%$, $H^2 = 63.18$			
Test of $\theta_i = \theta_j$: $Q(3) = 88.22$, $p = 0.00$			

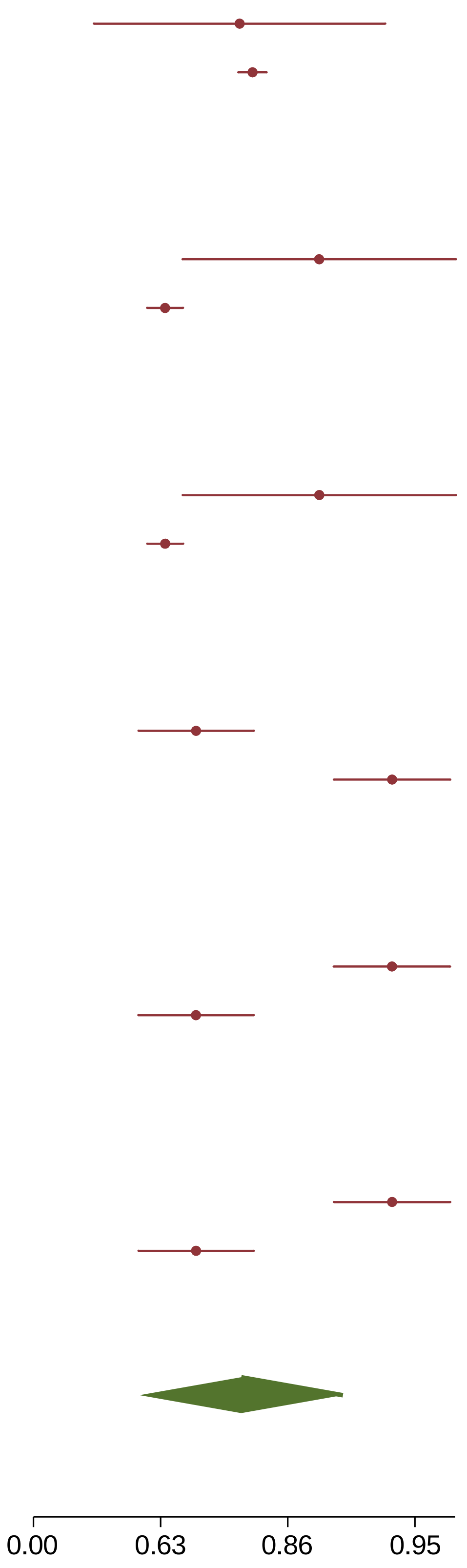

Random-effects REML model

Figure S6:The vaccine effectiveness against Delta variant for the death by variables in the unvaccinated control group

Study	K	Effectiveness with 95% CI	p-value
**time**			
1	6	0.90 [0.83, 0.94]	0.000
4	10	0.90 [0.80, 0.95]	0.000
8	12	0.81 [0.49, 0.93]	0.001
12	13	0.83 [0.51, 0.94]	0.001
16	7	0.62 [-0.04, 0.86]	0.060
20	4	0.71 [0.30, 0.88]	0.006
Test of group differences: Qb(5) = 9.18, p = 0.10			
**age**			
>=12 yr	28	0.82 [0.65, 0.91]	0.000
>50yr	24	0.85 [0.77, 0.91]	0.000
Test of group differences: Qb(1) = 0.23, p = 0.63			
**rob**			
low	50	0.84 [0.76, 0.89]	0.000
moderate	2	0.96 [0.96, 0.96]	0.000
Test of group differences: Qb(1) = 41.24, p = 0.00			
**booster**			
Any	6	0.93 [0.88, 0.96]	0.000
BNT162b2	43	0.81 [0.69, 0.88]	0.000
ChAdOx1	1	0.98 [0.97, 0.99]	0.000
CoronaVac	1	0.84 [0.77, 0.89]	0.000
mRNA	1	0.96 [0.86, 0.99]	0.000
Test of group differences: Qb(4) = 85.56, p = 0.00			
**studydesign**			
case control	4	0.94 [0.83, 0.98]	0.000
negative case control	10	0.96 [0.94, 0.98]	0.000
cohort	38	0.74 [0.60, 0.83]	0.000
Test of group differences: Qb(2) = 32.87, p = 0.00			
**previouscovidinfection**			
no	1	0.96 [0.86, 0.99]	0.000
yes	51	0.85 [0.77, 0.90]	0.000
Test of group differences: Qb(1) = 3.81, p = 0.05			
**Overall**		0.85 [0.78, 0.90]	0.000

Heterogeneity: $\tau^2 = 1.49$, $I^2 = 92.95\%$, $H^2 = 14.18$

Test of $\theta_i = \theta_j$: Q(51) = 532.31, p = 0.00

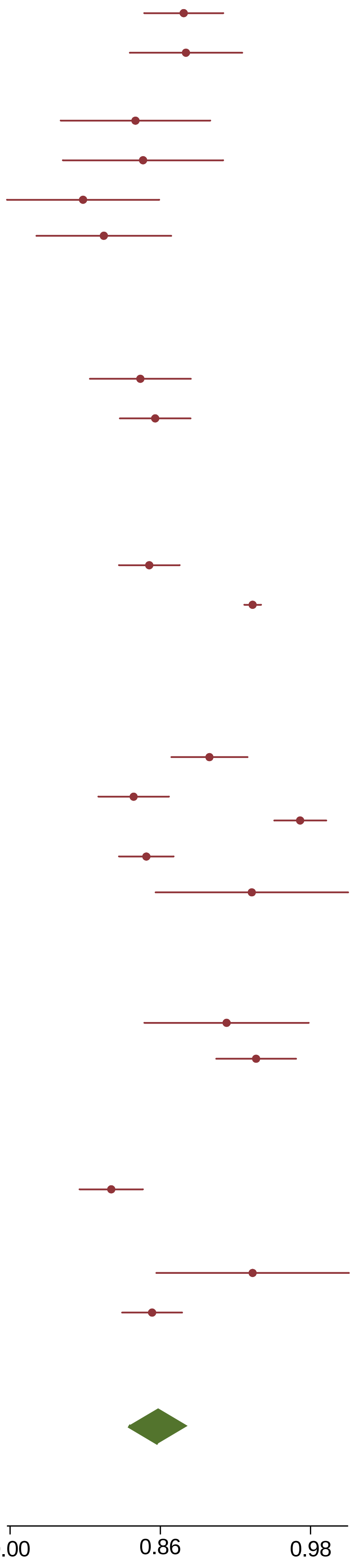

Random-effects REML model
